# Operative management of recurrent parastomal hernias after transversus abdominis release: a single-center experience

**DOI:** 10.1007/s00464-025-12214-1

**Published:** 2025-11-03

**Authors:** William C. Bennett, Pavel Lenkov, Kimberly P. Woo, Andrew S. Conner, Alvaro C. Carvalho, Kimberly S. Miles, Sara M. Maskal, Daphne Remulla, Ryan C. Ellis, Benjamin T. Miller, Lucas R. Beffa, David M. Krpata, Ajita S. Prabhu, Michael J. Rosen, Clayton C. Petro

**Affiliations:** 1 Cleveland Clinic Foundation, Cleveland, OH USA; 2 Case Western Reserve University School of Medicine, Cleveland, OH USA; 3 Northwestern Medical Center, Chicago, IL USA

**Keywords:** Parastomal hernia, Recurrent parastomal hernia, Retromuscular hernia repair, Colostomy, Ileostomy

## Abstract

**Introduction:**

Parastomal hernia recurrences after repair utilizing a transversus abdominis release (TAR) with retromuscular mesh presents a challenging clinical situation with little data available for guidance. This descriptive study characterizes our center’s experiences with operative management of recurrent parastomal hernias after TAR and associated outcomes.

**Methods:**

Open parastomal hernia repairs with TAR performed at our institution from August 2014 through August 2023 were identified via the Abdominal Core Health Quality Collaborative (ACHQC) database and reviewed to assess for instances of recurrence and reoperation. Electronic medical records were reviewed to confirm supplement operative and follow-up details. Hernia characteristics, operative characteristics, and outcomes of cases for these recurrent cases were abstracted and analyzed.

**Results:**

Following 172 open parastomal hernia repairs with TAR, 63 recurrences were identified, and 29 patients underwent 39 subsequent operations, as five patients required at least two reoperations for recurrence. Open redo retromuscular repair (25/39, 64%) was the most utilized operation, the most frequent final operation (23/29, 79%) and the open redo retromuscular Sugarbaker mesh orientation featured the lowest recurrence rate of ~ 5% at 1 year. All cases of primary repair or re-siting the stoma through previous retromuscular mesh required an additional operation for recurrence. Of the six patients (21%) who have avoided an open redo-RM repair, three have had a laparoscopic Sugarbaker, and two have had an open onlay.

**Conclusion:**

While all utilized techniques demonstrate the ability to temporize patients, most patients with parastomal hernia recurrence after a TAR ultimately require an open redo retromuscular repair which appears to be the most definitive option.

Parastomal hernia (PSH) is a common complication of stoma formation, and perhaps an inevitable outcome for permanent stomas given sufficient follow-up [1–3]. Symptomatic PSHs often require surgical repair due to diminished quality of life or increasing risk of surgical emergencies [4]. Many operative approaches have been suggested for repair [5–7] and/or prophylaxis [8, 9], and we have previously described our preference for open repair with posterior component separation (PCS) and transversus abdominis release (TAR) to accommodate wide retromuscular mesh reinforcement [10, 11]. The proliferation of such retromuscular techniques has led to improved rates of recurrence free survival [3, 12]. However, subsequent parastomal recurrences and operative management of those patients who have already had a TAR have received minimal attention in contemporary literature. This analysis describes and characterizes the operative management of recurrent PSHs (rPSH) after TAR at a single institution and aims to collate approaches and their outcomes.

## Methods

After receiving approval from the Institutional Review Board, the Abdominal Core Health Quality Collaborative (ACHQC) was queried for all patients aged ≥ 18 years who underwent open (including minimally invasive with conversion to open) repair of hernia with a parastomal component in a RM fashion with PCS and TAR at our institution from August 2014 to August 2023 and had registry documentation of recurrence via patient or clinician report. Patient-reported recurrence was defined as response of “Yes” to the question “Do you feel a bulge?” on the ventral hernia recurrence inventory (VHRI) questionnaire. The ACHQC registry is a prospectively designed international database to track hernia-specific operative features and outcomes. Participant surgeons enter preoperative and operative characteristics, and clinician and patient-reported postoperative outcomes are routinely tracked as standard of care. Identified records were exported and electronic medical record (EMR) abstraction was conducted to confirm all inclusion criteria were met: (1) patients underwent prior PCS with TAR for PSH at our center, (2) stoma was not reversed in the index operation nor subsequently, (3) recurrence was confirmed on imaging, and (4) patient underwent reoperation for recurrence at center with a member of the CCF Abdominal Wall Reconstruction practice group. The following exclusion criteria were simultaneously applied to identified cases: (1) urgent or emergent repair, (2) reoperation for reason other than hernia recurrence (though hernia repair during another indicated operation was allowed), or (3) hernia repair at an outside center. Hernias repaired non-emergently during admission for obstruction that subsequently resolved were included. Additional operations for recurrence identified during chart review, but not by the ACHQC query, were included. Only operations which had been conducted prior to final data collection on November 1, 2024 were included; operations scheduled after this date were not considered.

Recurrence on imaging was defined as Moreno-Matias class II/III (any intrabdominal viscera other than stoma-loop bowel present external to fascia) or documented clinician diagnosis of recurrence if physical exam or radiographic evidence was cited.

Additional patient characteristics and operative details for each case were collected by EMR review, including visible records from external organizations, including patient age, sex, American Society of Anesthesiologists class (ASA), body mass index (BMI, kg/m^2^), smoking status, modified hernia grade, operative history, dimensions of fascial defect, mesh type, mesh size, mesh position, operative approach, stoma disposition, and repair technique. Short-term outcomes obtained from EMR review included wound class and 30 day wound morbidity including surgical site infection (SSIs), surgical site occurrences (SSOs), and SSOs requiring procedural intervention (SSPOI). Defect length and width were recorded from the ACHQC or operative report when available, measurements reflect the European Hernia Society (EHS) incisional system measurement which importantly includes the lateral-most margin of the lateral-most defects for width (and cranial-caudal margins for length) [13]. Thus, defect dimensions in cases featuring concurrent midline hernias are not specific to the parastomal defect alone.

Time from reoperation to last known follow-up was collected for cases without known recurrence. Time until recurrence was instead documented for cases with known recurrence.

### Surgical approaches and categorization

Though all operative features were collected, operations were grouped as:

- open redo TAR PSH repair with a redo retromuscular dissection and new mesh placement (redo-RM)
- onlay
- intraperitoneal sublay
- primary
- relocation

Operations were defined as redo-RM if a repeat retromuscular dissection was done through a midline laparotomy and new mesh was placed in the retromuscular plane for reinforcement of the stoma site, midline laparotomy, and the prior stoma site (if relocation was also done). The orientation of the stoma/mesh interface was conducted in a retromuscular Sugarbaker (SB) or keyhole fashion as previously described [11, 14].

For onlay repairs, a vertical incision was made lateral or medial to the stoma, a cutdown to the anterior fascia was performed, and circumferential dissection of the hernia sac was completed before fascial closure. Next, a 15 × 15 cm medium-weight polypropylene mesh secured to the anterior fascia with staples and/or fibrin sealant. The uncut side of the mesh was placed medially, the tails were oriented laterally and closed with permanent suture around the stoma.

Intraperitoneal sublay operations were conducted in both open and laparoscopic fashions and mesh orientations are reported for each. Laparoscopic intraperitoneal sublays featured a SB configuration, and these operations include complete adhesiolysis and synthetic barrier-coated mesh fixation with tacks and trans-fascial sutures [15].

Repairs were defined as primary if the stoma was not relocated (either left in-situ or re-matured at the same site), the fascial defect was closed, and no new mesh was placed in an onlay or sublay position.

Repairs were defined as a primary relocation if the stoma was relocated in a keyhole fashion through previously placed retromuscular mesh, the fascial defect was closed, and no new mesh was placed in a sublay position.

Stoma disposition was characterized as being left in-situ, re-matured at the same site, or relocated. Any disruption of the mucocutaneous junction, including takedowns for bowel resection, was defined as re-maturation unless the operative report specified creation of a new trephine at an alternative site.

Outcomes of interest included 30-day wound morbidity defined as surgical site occurrence (SSO), surgical site infection (SSI), surgical site occurrence requiring procedural intervention (SSOPI), as well as recurrence and reoperation for recurrence.

### Statistical analysis

Descriptive statistics were generated to produce median and interquartile range (IQR). Comparisons of continuous variables between two groups utilized unpaired Welch’s t tests when data was normally distributed or Mann–Whitney test otherwise; continuous variable comparisons between three or more groups were conducted utilizing nonparametric Kruskal–Wallis analysis of variance testing followed by Dunn’s multiple comparisons tests with GraphPad Prism version 10.4.1 for macOS (GraphPad Software; Boston, MA; www.graphpad.com). Categorical comparisons across two or more groups were conducted via Chi-Square test of independence; and Fisher’s exact test was used in lieu of Chi-Square test for groups with fewer than 5 subjects with STATA 16 IC (StataCorp. 2023. Stata Statistical Software: Release 16. College Station, TX: StataCorp LLC.). Statistical significance was predetermined at *P*-value < 0.05.

## Results

Following exclusions, 172 ACHQC records were identified as having had an open parastomal hernia repair with TAR and retromuscular mesh between August 2014 and August 2023 with follow-up data indicative of possible recurrence. At a median follow-up of 2 years (IQR 415–1301 days), 63 radiographic recurrences were identified (representing 53 patients) following review of each patient’s medical record. Of the 53 patients, 29 patients (55%) underwent at least one elective reoperation for recurrence at our center. Ultimately, 39 reoperations for recurrence at our institution from August 2014 to November 2024 were identified and included in this study, as nine patients underwent more than one reoperation with an eligible surgeon. Demographics for patients who underwent reoperation at each instance of reoperation and patients who did not undergo reoperation are reported in Table 1. Briefly, 68% of patients had undergone more than one previous repair, and reoperations for recurrence occurred at a median of 19 months (IQR 11–13) from their last open retromuscular TAR repair. The most cited indications for reoperation were intermittent obstruction (41%) and severe impairment of activities (46%); conversely a majority of non-reoperative patients (71%) were documented as asymptomatic at time of recurrence diagnosis. Baseline characteristics were grossly similar between groups; however, non-reoperative patients tended to have higher ASA class, fewer prior hernia repairs, and end colostomies (rather than ileal conduits or end ileostomies) — Table 1.

**Table 1:** Characteristics of recurrent patients who did vs did not undergo subsequent repair

	Reoperation		Non-reoperation		
Characteristic	*n* = 39		*n* = 24		*p*
Age	61	(52, 70)	65	(58, 70)	0.45
Female gender	17	44%	8	33%	0.44
BMI	31.4	(25.3, 34.8)	31.2	(27.3, 35.3)	0.77
HTN	22	56%	18	75%	0.18
DM	8	21%	4	17%	1
COPD	5	13%	3	13%	1
Current nicotine use	6	15%	1	4%	0.24
History of cancer	10	26%	11	46%	0.11
History of inflammatory bowel disease	13	33%	6	25%	0.58
ASA class					
2	34	87%	5	21%	0.0001
3	5	13%	19	79%	
Number of prior hernia repairs					
1	7	18%	13	55%	0.008
2	11	28%	8	33%	
3	9	23%	1	4%	
4	7	18%	2	8%	
5	5	13%	0	0%	
Stoma type					
EI	20	51%	9	38%	0.027
EC	6	15%	11	46%	
Ileal conduit	13	33%	4	16%	
Prior RM mesh orientation*					
SB	8	28%	10	42%	0.28
KH	21	72%	14	58%	
Months to reoperation	19	(11, 33)	–	–	
Primary symptom					
Obstruction	16	41%	0	0	0.0003
Fistula	1	3%	0	0	0.4
Activity interference	18	46%	0	0	< 0.0001
Pain	4	10%	1	4%	0.39
Nausea	0	0%	1	4%	0.2
Asymptomatic	0	0%	17	71%	< 0.0001
Not captured	0	0%	5	21%	–

^*^For reoperative patients, only the initial retromuscular repair mesh orientation is reported (as such *n* = 29)

Of the 29 patients who underwent reoperation for parastomal hernia recurrence after a previous open retromuscular TAR repair, 17 initially underwent an open redo TAR parastomal hernia repair with a new retromuscular dissection and new retromuscular mesh placement (redo-RM) (Table 2). Furthermore, three of those patients required yet another second open redo-RM repair (3rd RM repair), one with an open intraperitoneal keyhole mesh placement done between those cases that promptly failed; this patient is scheduled for a third open redo-RM repair. Complete excision of previously placed mesh was conducted in two of these cases.

**Table 2:** Operative characteristics stratified by parastomal hernia repair approach for reoperations

	All		Re-do RM		Onlay		IP		Primary		Relocate	
	*n* =	39	*n* =	25	*n* =	5	*n* =	4	*n* =	3	*n* =	2
EHS defect width (cm)*	13	(4, 19.75)	15	(13, 22)	4	(3, 4)	4.5	(3.75, 5)	4	(3.75, 12.5)	4	(4, 4)
EHS defect length (cm)	15	(4, 24.5)	23	(16, 13)	3	(3, 3)	4	(3.75, 4)	6	(4.75, 10.5)	4	(4, 4)
Concurrent midline hernia present	17	44%	17	68%	0	0%	0	0%	0	0%	0	0%
PSH defect width (cm)**	–	–	22 (n = 5)	(19, 26)	–	–	–	–	–	–	–	–
PSH defect length (cm)	–	–	16 (n = 5)	(15, 20)	–	–	–	–	–	–	–	–
OR time												
0–59 m	1	3%	0	0%	1	20%	0	0%	0	0%	0	0%
60–119 m	5	13%	0	0%	3	60%	1	25%	1	33%	0	0%
120–179 m	9	23%	4	16%	1	20%	2	50%	0	0%	2	100%
180–239 m	10	26%	7	28%	0	0%	1	25%	2	67%	0	0%
240 + m	14	36%	14	56%	0	0%	0	0%	0	0%	0	0%
Stoma disposition												
Left in-situ	17	44%	5	20%	5	100%	4	100%	2	67%	–	–
Rematured at same site	6	15%	6	24%	0	0%	0	0%	1	33%	–	–
Relocated	16	41%	14	56%	0	0%	0	0%	0	0%	2	100%
Sublay technique												
Sugarbaker	22	56%	19	76%	–	–	3	75%	–	–	–	–
Keyhole	9	23%	6	24%	–	–	1	25%	–	–	–	–

^*^European Hernia Society incisional hernia measurement includes total length from lateral-most extents of the left-most and right-most defects, as such these contain measurements do not pertain exclusively to the parastomal hernia

^**^These measurements are from cases in which no midline hernia was explicitly reported and length/width were documented, as such, the defect dimensions regard only the parastomal component

Overall, prior mesh was completely removed in three cases and partially excised in another three additional cases. In one instance, prior retromuscular mesh was removed and replaced with a new retromuscular mesh in a keyhole configuration, and no mesh-viscera bowel interactions were noted. In two redo-RM Sugarbaker cases, complete excision of previously placed intraperitoneal mesh was done due to mesh-bowel interactions, but the prior retromuscular mesh was left in-situ. Partial mesh excision of prior retromuscular prostheses was documented three ties. In one instance a third of the prior retromuscular mesh was removed due to bowel interactions and in another the mesh was removed given concern for mesh-bladder interaction. Each of these patients received bilateral redo-RM repair with mesh in a Sugarbaker configuration. In the third partial retromuscular mesh excision, no mesh-viscera interactions were noted, the operative report notes the mesh was dissected off the posterior sheath and excised until further lateral dissection threatened the integrity of the posterior sheath.

The next most common initial approach included five attempts of mesh onlay, of which four subsequently recurred at 4, 9, 21, and 34 months; notably, the fifth repair was lost to follow-up after a 20-day post-operative visit. Three of the known recurrences went on to undergo an open redo-RM repair. The fourth recurrence did not pursue repeat operative repair.

Three patients initially underwent a primary repair. One recurred at 110 days, and underwent a laparoscopic Sugarbaker repair, and is now scheduled for an open redo-RM repair. The other two underwent reoperations with surgeons outside of our practice group and were not further tracked. One of these recurred at 42 days and a laparoscopic repair was attempted, but this was converted into an open redo-RM repair with a keyhole configuration. The third patient was found to recur at 203 days, and subsequently underwent stoma relocation through previous retromuscular mesh, without new retromuscular dissection or new mesh placement.

Two patients who initially had a reoperation with stoma relocation through previous retromuscular mesh but without an open redo-RM repair. Both ultimately recurred, at 92 and 294 days, and subsequently underwent open redo-RM repairs.

Finally, two patients initially received a laparoscopic Sugarbaker repair without fascial closure and have not required subsequent revision. Mean follow-up of these two cases was 81.5 days. The previously mentioned laparoscopic Sugarbaker repair performed after failed primary repair was found to have recurred at 992 days.

**Fig. 1:**
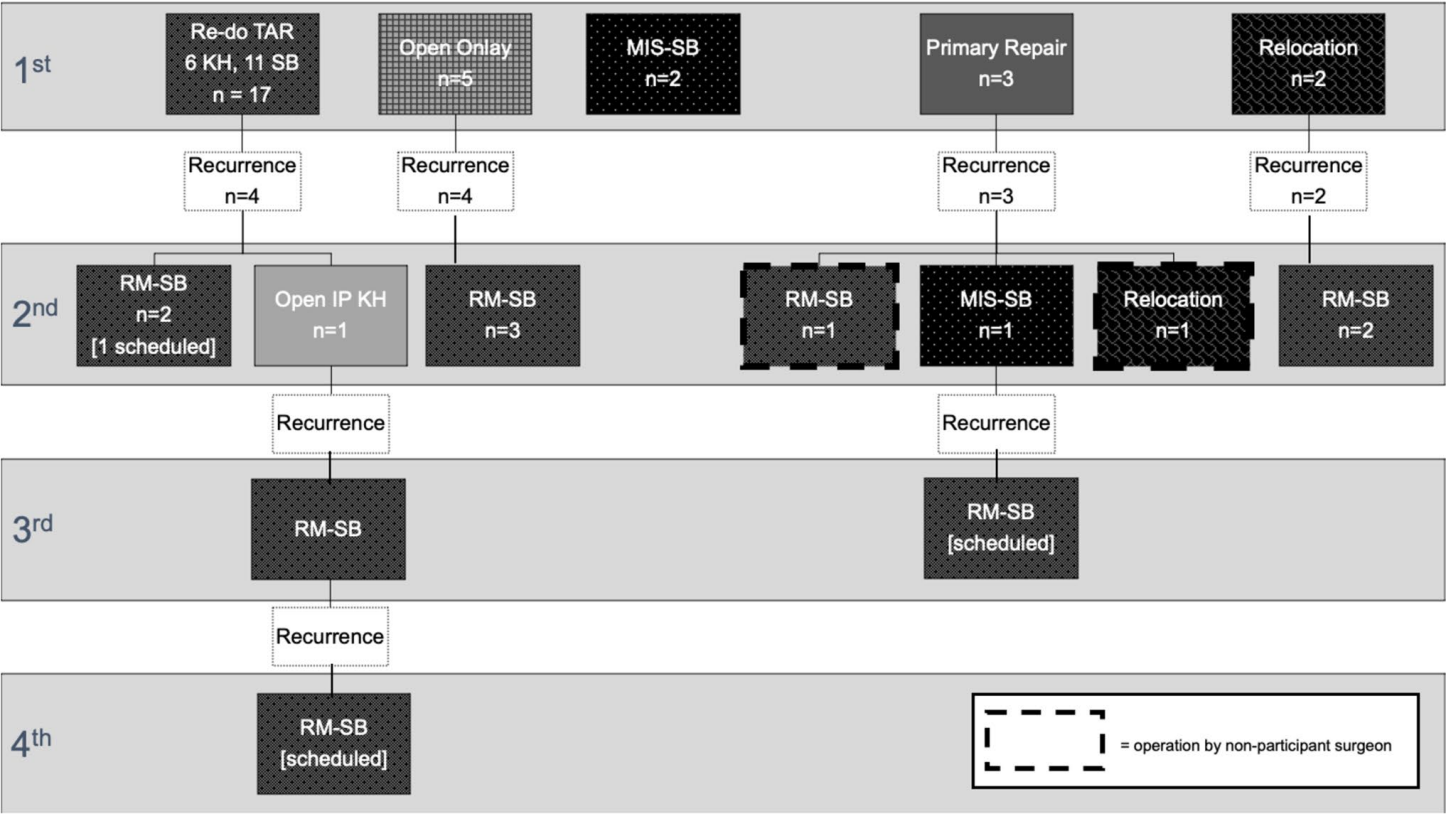
Operative progression of patients undergoing iterative repairs of recurrent parastomal hernias after initial repair with TAR. *RM-SB* retromuscular Sugarbaker, *MIS-SB* minimally invasive Sugarbaker, *IP SB* Intraperitoneal Sugarbaker, *Redo TAR* redo transversus abdominus release, *RM KH* retromuscular keyhole

To summarize (Fig. 1), 23/29 (79%) patients ultimately went on to get at least one open redo TAR parastomal hernia repair with a redo-RM dissection and new mesh placement. A total of 25 redo TAR parastomal hernia repairs were done by our group, 19 in a retromuscular Sugarbaker mesh configuration and six in a keyhole orientation. Three of the retromuscular Sugarbaker cases and three of the retromuscular keyhole cases were assigned via randomization during a previously published randomized trial [11]. In those 25 open redo-RM cases, 14 (56%) had their stoma relocated, five (20%) had their stoma left in situ, and six (24%) had the stoma mobilized and re-matured at the same trephine. There was no difference in stoma disposition between open redo-RM Sugarbaker and redo-RM keyhole cases (*p* = 0.66).

Of note, all utilizations of mesh for reinforcement were medium-weight polypropylene, with the exception of one redo-RM repair in which case a biologic mesh was utilized per randomization in the context of a clinical trial [16]. All patients who underwent primary repair or stoma relocation without new mesh placement ultimately required another operation. Of the five patients (17%) who have avoided an open redo-RM repair, three have had a laparoscopic Sugarbaker, and two have had an open onlay. One of these open onlays did recur but underwent one of the aforementioned laparoscopic Sugarbaker repairs.

### 30-day outcomes and long-term recurrence

Wound morbidity and 30-day outcomes are summarized in Table 3. Total 30-day rates of SSO, SSI, and SSOPI were 31%, 26%, and 13%, respectively. Small group sizes limit meaningful comparisons of technique. While the recurrence rate for all patients was 33% at a median follow-up of 9 months, the most commonly utilized approach of open redo-RM repair had the lowest recurrence rate of 15% at a median follow-up of approximately one year.

**Table 3:** Thirty day surgical outcomes and long-term recurrence follow-up

	All		Re-do RM		Onlay		IP		Primary		Relocate	
	*n* =	39	*n* =	25	*n* =	5	*n* =	4	*n* =	3	*n* =	2
30 day SSO	12	31%	8	32%	2	40%	1	25%	0	0%	1	50%
30 day SSI	10	26%	6	24%	2	40%	1	25%	0	0%	1	50%
30 day SSOPI	5	132%	3	12%	1	20%	1	25%	0	0%	0	0%
30 day reoperation	4	10%	2	8%	1	20%	1	25%	0	0%	0	0%
Median time of last follow-up, days (IQR)	307	(58.5, 667.75)	384	(70, 691)	20	(20, 20)	81.5	(75.75, 87.25)	-	(-, -)	-	(-, -)
Recurrence	15	39%	4	16%	4	80%	2	50%	3	100%	2	100%
Median time to recurrence, days (IQR)	297	(163, 704.5)	414.5	(294.75, 681)	481	(253.5, 761)	868	(806, 930)	110	(76, 156.5)	193	(142.5, 243.5)

One recurrence was observed after open redo-RM Sugarbaker for a 5% recurrence rate and median follow-up time was 384 postoperative days (IQR 47, 659.5) when considering last date of follow-up (for non-recurrence) or first date of recurrence. Three recurrences were identified in the open redo-RM keyhole group (50%) at a median follow-up time of 508 days (IQR 257, 765). The difference between recurrence rates of RM Sugarbaker and RM keyhole repairs in this context was statistically significant (*p* = 0.018); length of follow-up, however, was not statistically different between groups (*p* = 0.69).

## Discussion

In this descriptive analysis of a single-center’s experience with operative management of parastomal hernia recurrences after previous open parastomal hernia repair with TAR and retromuscular mesh, we demonstrate the utilization of myriad surgical approaches for these challenging patients. Overall, 55% of imaging-identified recurrences underwent at least one reoperation; of these, most ultimately required an open redo-RM dissection with placement of additional retromuscular mesh, which also appears to be the most durable option. Recurrence was specifically lowest for patients who underwent an open redo-RM repair in a Sugarbaker orientation (5% at ~ 1 year). Other approaches remain feasible and safe for temporization with occasional long-term success. That 31% of reoperative patients required more than one additional operation for recurrence suggests definitive PSH management remains elusive and in desperate need of further innovation.

Scant literature considers rPSH in isolation, though they appear to be clinically distinct entities compared to their primary counterparts with greater wound morbidity, greater recurrence, and decreased time between repair and recurrence [3, 17]. Three descriptive series of rPSH repair have been published; however, these concern patients who underwent previously minimally invasive Sugarbaker repair with intraperitoneal mesh [18, 19] or initial repair with a specific mesh product [20]. Bloemendaal et al. [19] described robotic RM-PCS as a viable option for primary and rPSH, however, in this case series of robotic RM-PCS, the RM plane had not previously been dissected and the technique was only used for the recurrence, not the primary hernia. While recurrent PSH-specific literature is less common than studies considering primary PSH, some studies consider both. Keller et al. [21] reported a series of open (featuring some RM-PCS) vs laparoscopic repairs which included primary and recurrent PSH, but neither adjusted nor controlled for recurrent status when considering adverse outcomes. This effort specifically represents the first attempt to describe operative approaches and short-term outcomes for parastomal hernia recurrences following a previous TAR. Here, we show that re-dissection of the retromuscular plane and new mesh placement for these patients is safe and feasible.

A preference for retromuscular Sugarbaker vs keyhole was seemingly demonstrated, particularly when repeat open redo-RM dissection was done. Some studies have suggested the keyhole to have associations with recurrence; however, our previous prospective randomized trial [11] identified no statistical difference in recurrence at 2 years (17% in RM Sugarbaker repairs vs 24% in RM keyhole repairs) when patients who were eligible for either repair were randomized, as have other contemporary investigations [11, 22]. Importantly, Sugarbaker repairs in that clinical trial showed an early recurrence benefit at the 1-year follow-up interval (8% vs 21.3% for keyhole repairs; *p* = 0.04). This is consistent with the findings in this project which demonstrated a 5% rate of recurrence for redo-RM Sugarbaker repairs at a median follow-up of ~ 1 year. Our data demonstrates a statistical difference between the 5% recurrence rate of redo-RM Sugarbaker repairs and the 50% recurrence rate observed in the keyhole group, however, consideration of follow-up duration and limited sample sizes are necessary when interpreting this finding. Statistical difference was not observed between the groups’ follow-up durations, but from a practical standpoint they cannot be deemed equivalent. Seven of the 19 (37%) patients in the redo-RM Sugarbaker group had a final follow-up less than 90 days and were functionally lost-to-follow-up, whereas the shortest follow-up interval for keyhole patients was 204 days. If even one of the lost-to-follow-up redo-RM Sugarbaker recipients experienced recurrence, the difference between recurrence rates would lose significance (fragility index =  + 1). The apparently lower rate of recurrence observed could well be a signal indicating a benefit of the Sugarbaker mesh configuration in the specific instance of redo-RM repair, but improved follow-up and expanded sample sizes would be needed to substantiate such a claim. Overall, the favorable recurrence profile for redo-RM Sugarbaker mesh orientation might be tempered with longer follow-up.

Stoma relocation was conducted intermittently and was patient-dependent, with decisions to relocate often predicated upon patient request or surgeon discretion. Literature is unresolved on differentiable rates of wound morbidity or PSH recurrence between relocation vs non-relocation of stomas, and the data presented here demonstrate no preference for one approach or the other [4, 7, 23, 24]. Further, no difference was observed in the stoma relocation rate (vs re-maturation at the same site or leaving stoma in situ) between the redo-RM Sugarbaker and keyhole configurations (53% vs 66%, respectively; *p* = 0.66). Similarly, decisions to excise previous mesh were intermittent and patient-dependent. Our description of surgical decision making in each instance is limited given the retrospective nature of this project; however, the standard practice in our group is to remove previously placed mesh when possible. Notably, for redo-RM repairs, it is often not possible or beneficial to remove all previous mesh. Of the three complete mesh excisions identified in our record review, two of them removed previously placed intraperitoneal mesh (one Gore-Tex and one unidentified). In the only documented total resection of retromuscular mesh, the surgeon’s report noted a favorable dissection plane facilitating total resection. In one of the identified partial excisions of retromuscular mesh, the surgeon similarly noted mesh was excised while the dissection was favorable but abandoned when further dissection threatened posterior sheath integrity. When mesh-viscera interactions were noted, the mesh was partially excised. Overall, decisions to relocate stomas and/or excise mesh are patient-dependent and multi-factorial. Foremost of these factors are the patients’ preferences for stoma position and if mesh-bowel/anatomy interfaces necessitate resection (or preservation) for safe dissection.

While our findings overall support the utilization of open redo-RM repair, MIS-SB appears to merit some consideration when feasible, particularly for those with a small defect and no concurrent midline incisional hernia. In the aforementioned study by Keller et al. [21], retrospective review of primary and recurrent PSH cases suggested MIS-PSH repair can delay open intervention [21]. They compared 31 patients who underwent MIS-SB vs 31 patients who underwent open repair, including 20 who underwent PCS. While the aim was not to analyze recurrent PSH, 43% of cases were for recurrent or multi-recurrent PSH. MIS repairs were associated with decreased operative time (55% of MIS cases took < 180 min vs 52% of open taking > 240 min, *p* < 0.001), length of stay (3 vs 7 days, *p* < 0.001), and dehiscence (3% vs 29%, p = 0.012). Further, recurrence-free survival was 79% for MIS-SB at 3 years vs 36% for open repairs, but 1/3rd of the open repairs were onlays. A second analysis was conducted which considered laparoscopic procedures vs open onlays and open sublays. While the open sublay group included one non-RM repair, the majority utilized at least one component separation. They identified multiple adverse associations with open sublay vs MIS-SB including SSO (48% vs 27%, *p* = 0.036) and length of stay (5 days vs 3, *p* < 0.01). Recurrence-free survival was 83% for IP mesh vs 42% for other sublay positions. Their results, notably, included patients with 2 or more prior PSH repairs and 88% of these underwent open reoperation. Overall, 71% of MIS-SB patients were non-recurrent vs 42% in open operations. Further, bioresorbable mesh was used in 50% of open cases, but synthetic mesh was implemented in 87% of laparoscopic cases, further confounding comparison of our results.

Nevertheless, the lone documented MIS-SB recurrence in our study occurred at 2.7 years postoperatively. This patient is presently scheduled to undergo redo-RM repair, but at nearly 7.5 years after MIS-SB. This progression may support a step-up approach from MIS-SB to redo-RM repair for patients who are technically amenable to that approach. While recurrence occurred within 3 years, symptom severity was limited and delayed reoperation for a notable period. Limited follow-up duration for the other MIS-SB patients (70 and 90 days) precludes anything but speculation from this data. However, this is a signal warranting further attention and consideration of a pathway incorporating both MIS-SB and open redo-RM repair.

Conversely, our data do not support a role for onlay or primary repairs of recurrent parastomal hernias after RM-PCS if definitive treatment is desired, with recurrence rates of 80% and 100%, respectively, all within nine months. However, it should be noted that while these repairs all met inclusion criteria (specifically, elective scheduling status), it cannot be said the repairs occurred under ideal conditions. Our design made no distinction between outpatient and inpatient elective cases. Two of the three primary repairs occurred during a patient’s admission for obstruction, though after resolution of obstruction. One primary repair occurred in the outpatient elective setting. This patient preferred to avoid open redo-RM surgery and a primary repair was offered with the patient aware of the high risk of recurrence. This patient ultimately underwent MIS-SB at 7 months. Of note, this is the MIS-SB recurrence who is now scheduled to undergo open redo-RM in the coming months. If a step-up approach is developed, the inclusion of primary repair prior to MIS-SB should perhaps be offered only with sufficient counselling, and perhaps not considered at all.

Only one patient in the onlay group underwent ‘elective’ repair during an inpatient admission following bowel obstruction. The other four were scheduled in an elective, outpatient fashion. For each onlay case, operative planning documentation noted a small bulge sensation reported by patients or a known small hernia defect on imaging, for which onlay was felt to represent a reasonable option.

Overall, our findings are important descriptions of reoperative approaches for patients who recur at their stoma site after previous retromuscular repair—an increasingly utilized technique, especially with descriptions of minimally invasive approaches [25–28]. While most literature suggests improved outcomes for retromuscular repairs, some degree of recurrence appears inevitable, as is the need for repair in some cases. While our results may no doubt inform surgeons facing the need for reoperative repair of PSH following prior PCS, our data must be considered in light of multiple limitations. First, all cases are from a high-volume abdominal wall reconstruction practice wherein open redo-RM repairs are frequently performed. Our inclusion criteria and search strategy exclusively identified patients who underwent TAR within our center and had previously documented concern for recurrence: patient-reported bulge sensation, physical exam documentation of a bulge, or imaging-proven recurrence. These criteria were set to ensure patients actually had a prior retromuscular dissection in the targeted planes, as reports of plane misidentification have been described [29, 30]. However, this limited generalizability in multiple ways. First, this underreported the number of repairs at our center for recurrent parastomal hernia after previous TAR; a number of operations were not captured via our search criteria due to this factor. For instance, if a patient presented to our center for repair of a recurrent parastomal hernia after TAR at another institution (and only received a non-TAR repair from our group), they would not have been captured on our query of the ACHQC. Second, the threshold to proceed with repeat open redo-RM repair for recurrent parastomal hernia is likely lower in our practice. This is not only true due to the abdominal wall reconstruction volume at our center, but the immediate availability of Urology and Colorectal services for reconstruction of conduits and stomas. The relationship is reciprocal, many of the cases included in this review saw Urology and Colorectal surgeons request assistance in parastomal hernia repair during existing plans to revise or relocate a dysfunctional stoma. Third, the search query’s original cohort of 172 records cannot be utilized to calculate a recurrence rate when considering the 70 records with imaging-confirmed recurrences. Not all patients in the cohort of 172 records had available imaging, duplicates of patients were identified, some cases were identified during electronic medical record review, and the search query excluded patients without known concern for recurrence. At best, our data capture strategy could be utilized to infer that 55% (29 patients out of 53 patients) with imaging-confirmed parastomal hernia recurrence after TAR will ultimately undergo at least one additional elective hernia repair operation; but we cannot attribute differential risk of recurrence or reoperation to features of their index operations. Finally, as our primary aim was not to capture reoperative decision-making, we also disregarded parastomal hernia classification. Parastomal hernias can be classified in a number of manners, but this analysis was agnostic to them [31, 32]. While classification systems have demonstrated clinical significance in operative planning purposes in primary PSH, contributing to that discussion was not the goal of this descriptive effort [33]. Future analyses featuring the cases identified in this effort may provide greater context into the decisions regarding operative approaches for these challenging patients.

## Conclusion

This descriptive study demonstrates a variety of operative approaches for recurrent parastomal hernia after prior open TAR at a high-volume abdominal wall reconstruction center demonstrating high utilization open redo-RM repair. Repairs appear to be most durable with a RM Sugarbaker mesh orientation, but discrepancies in sample size and follow-up duration across techniques encourage further investigation. While other approaches are suitable for temporizing patients, we demonstrate open redo-RM dissection is a safe, feasible, and durable surgical option for parastomal recurrences following prior TAR.

## Abbreviations

TAR: Transversus abdominus release
PCS: Posterior component separation
RM: Retromuscular
SB: Sugarbaker
KH: Keyhole
PSH: Parastomal hernia
rPSH: Recurrent parastaomal hernia
